# Safety and Tolerability of the 1440‐ and 1927‐nm Non‐Ablative Fractional Diode Laser System for Skin Resurfacing: A Review of Current Literature

**DOI:** 10.1111/jocd.70523

**Published:** 2025-11-12

**Authors:** Roy G. Geronemus, Jordan V. Wang, Abby A. Jacobson, Ellen S. Marmur, Kristel D. Polder

**Affiliations:** ^1^ New York University Medical Center New York New York USA; ^2^ Laser & Skin Surgery Center of Pennsylvania Devon Pennsylvania USA; ^3^ Bausch Health Companies Inc Bridgewater New Jersey USA; ^4^ Marmur Medical New York New York USA; ^5^ Dallas Center for Dermatology and Aesthetics Dallas Texas USA

**Keywords:** 1440 nm, 1927 nm, diode, fractional, laser, non‐ablative, safety

## Abstract

**Background:**

Energy‐based devices, such as lasers, provide effective treatments for skin resurfacing. Ablative fractional lasers have a higher risk of adverse events (AEs), like scarring and postinflammatory hyperpigmentation, particularly in patients with darker skin types, than non‐ablative fractional lasers. The 1440‐ and 1927‐nm non‐ablative fractional diode laser (NFDL) system is indicated for use in dermatological procedures requiring the coagulation of soft tissue and for general skin resurfacing procedures.

**Aim:**

To help inform clinical decision‐making about the dual 1440/1927‐nm NFDL system, particularly for treating patients with diverse skin types who require safe and effective resurfacing treatments.

**Methods:**

A PubMed search of literature was conducted to review safety and tolerability outcomes from clinical studies of 1440‐ and 1927‐nm NFDL treatments.

**Results:**

Expected skin reactions, including erythema, edema, and crusting, were mild to moderate and self‐limited for concurrent and individual use of the 1440‐ and 1927‐nm handpieces. Mild discomfort and heat sensation, which were also expected, indicated that treatment was well tolerated. Safety was also demonstrated in patients with skin of color, with no serious AEs. Levels of patient satisfaction were high.

**Conclusions:**

The dual 1440/1927‐nm NFDL system is a safe and well‐tolerated option for resurfacing of diverse skin types with minimal postprocedural downtime and reduced risk of AEs relative to ablative lasers.

## 1 Introduction

**FIGURE 1:**
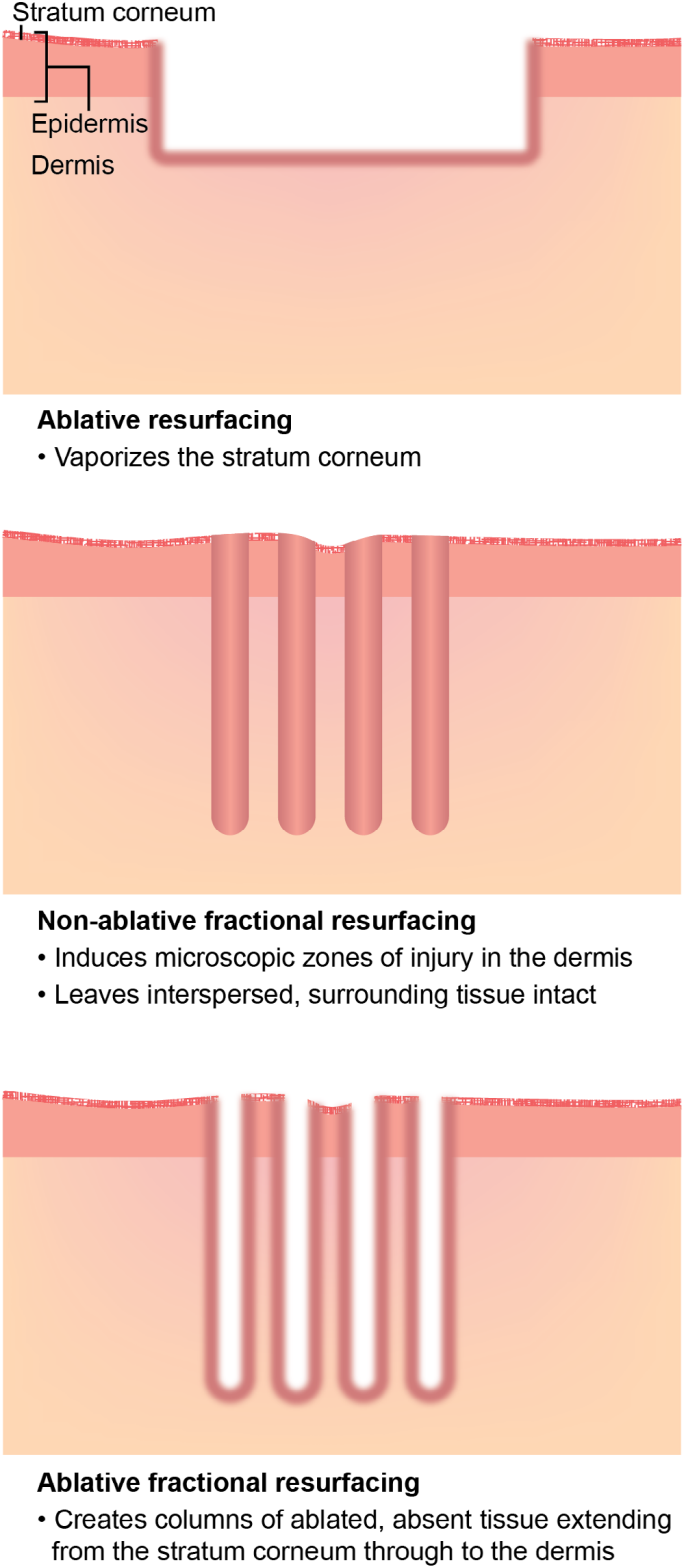
Non‐ablative fractional skin resurfacing compared to other laser technologies [2, 5, 6].

Energy‐based skin resurfacing devices deliver thermal energy to tissue to stimulate neocollagenesis and wound‐healing responses [1]. While ablative fractional lasers can be effective, they vaporize the stratum corneum, require reepithelialization, and are associated with substantial postprocedural downtime and risks of postinflammatory hyperpigmentation (PIH) and scarring [1, 2, 3, 4]. This makes them unsuitable for many patients, particularly those with darker Fitzpatrick skin types (FSTs) [4]. Conversely, non‐ablative fractional lasers, which operate at mid‐infrared wavelengths from 1320 to 1927 nm, provide gentle resurfacing and coagulate tissue in interspersed microscopic zones without damaging the skin surface (Figure 1) [2, 5, 6].

Fractional photothermolysis is an approach to skin resurfacing where the laser produces a pixelated array of light energy that creates microscopic columns of thermally damaged skin (termed microscopic treatment zones [MTZs]) surrounded by undamaged tissue [6, 7]. Immediately after treatment, epidermal and dermal cells undergo necrosis within MTZs [7]. Twenty‐four hours after treatment, continued loss of dermal cell viability coincides with loss of collagen; epidermal defects are repaired via keratinocyte migration [7, 8]; and damaged dermal content is incorporated into microscopic epidermal necrotic debris (MENDs), shuttled through the epidermis, and exfoliated from the surface [9]. At 14 days, synthesis and deposition of dermal collagen are observed [10], with complete replacement of thermally damaged collagen after 3 months [8]. This approach provides effective skin resurfacing with reduced postprocedural downtime and adverse event (AE) risk relative to ablative fractional lasers, thereby improving treatment accessibility for a wider patient population [11].

**FIGURE 2:**
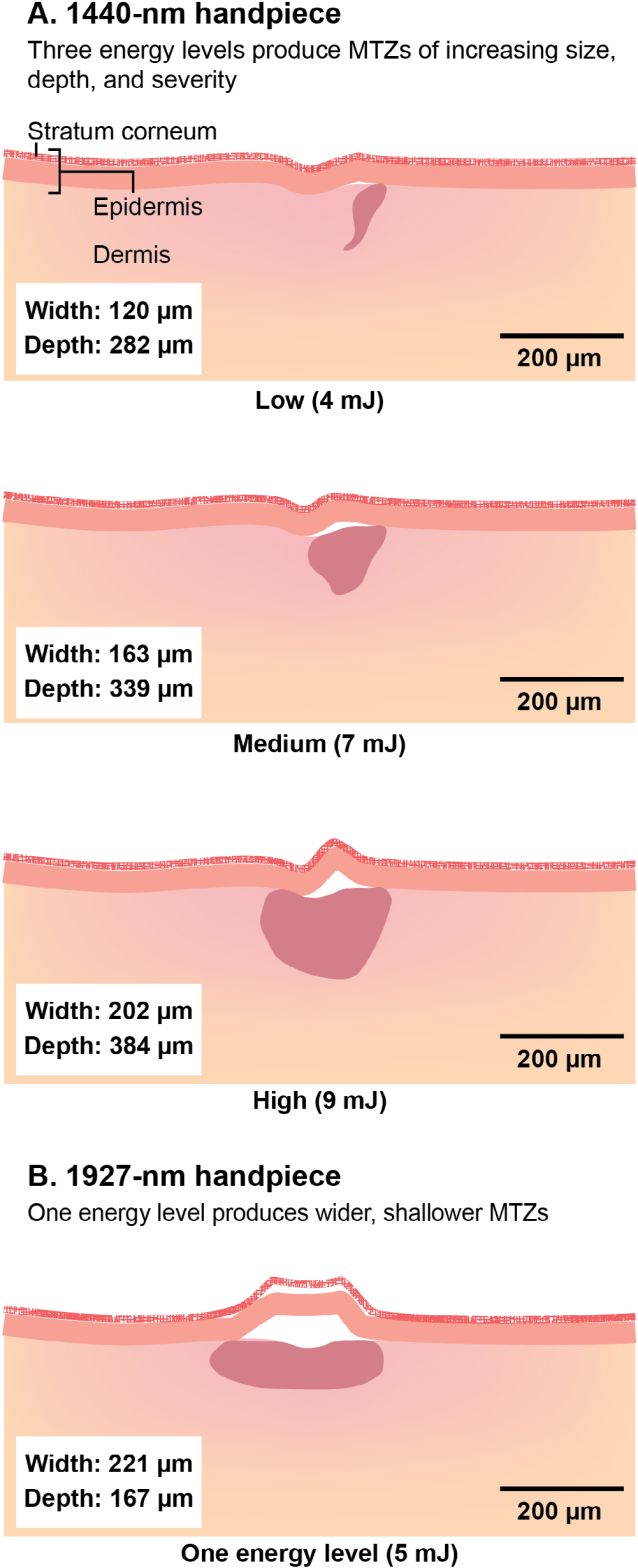
Microscopic treatment zones (MTZs) produced by the (A) 1440‐nm and (B) 1927‐nm handpieces [5, 10, 13, 14]. MTZ measurements were obtained from abdominal skin grafts [5, 13].

The 1440‐ and 1927‐nm non‐ablative fractional diode laser (NFDL) system with Clear+ Brilliant 1440‐nm laser (Solta Medical) and Perméa 1927‐nm laser (Solta Medical) is indicated for use in dermatological procedures requiring the coagulation of soft tissue and for general skin resurfacing procedures (specifications are listed in Table 1) [5, 12]. The 1440‐nm handpiece of the dual 1440/1927‐nm NFDL system offers 3 increasing energy levels to produce a dose‐dependent response in MTZ size, penetration depth, and severity (Figure 2A) [5, 10, 13]. Additionally, the 1927‐nm wavelength grants a higher absorption coefficient for water compared to the 1440‐nm wavelength, generating wider and shallower MTZs (Figure 2B) [5, 13, 14] that disrupt the epidermis to promote superficial resurfacing of the skin [14]. The dual 1440/1927‐nm NFDL system provides gentle skin resurfacing with improved patient comfort and minimal downtime [6, 11], which may be ideal for patients seeking low‐discomfort treatments and those with darker skin types.

**TABLE 1:** Technical specifications for individual handpieces of the dual 1440/1927‐nm NFDL system [5, 12].

Specification	1440‐nm handpiece	1927‐nm handpiece
Wavelength, nm	1440 ± 20	1927 ± 20
Maximum power (average), W	2.5	0.9
Maximum pulse energy, mJ	9	5
Maximum pulse width, ms	< 5	5
Pulse repetition rate, Hz	< 400	< 150

Fixed energy levels^a^	Low	Medium	High	Low	Medium	High
Depth, μm	280	340	390	170	170	170
Energy, mJ	4	7	9	5	5	5
Coverage, %	2	3.5	4.5	2.5	3.75	5

Abbreviation: NFDL, non‐ablative fractional diode laser.

^a^ Specifications are based on four total passes.

To help inform clinical decision‐making, particularly for treating patients with diverse skin types who require safe and effective resurfacing treatments, this review discusses the mechanism of action of the dual 1440/1927‐nm NFDL system and summarizes studies reporting safety and tolerability outcomes of 1440‐ and 1927‐nm NFDL treatments for skin resurfacing.

## 2 Materials and Methods

A PubMed search of literature was conducted using the following search terms: “non‐ablative fractional laser,” “fractional,” “fractionated,” “laser,” “1440,” and “1927,” from 2011 to 2024. A total of 68 articles were identified. Of these, 11 randomized trials and cohort studies that examined safety outcomes of 1440‐ and 1927‐nm NFDL treatments were included. Case reports, reviews, split‐face studies, studies without safety outcomes, and those that did not examine 1440‐ and 1927‐nm NFDL treatments were excluded, as were non–English language studies.

## 3 Results

### 3.1 Safety and Tolerability

Overall, seven studies were identified that reported safety and tolerability of 1440‐ and 1927‐nm NFDL treatments and included FSTs I to VI (Table 2) [14, 15, 16, 17, 18, 19, 20].

**TABLE 2:** Studies investigating the safety and tolerability of the dual 1440/1927‐nm NFDL.

Author, year	Study design	Population (*N*), FST	Study procedures	Safety and tolerability outcomes^a^
Dual 1440/1927‐nm NFDL				
Hoffman, 2024 [15]	Prospective, single‐arm, nonrandomized	Female adults with photodamage to their neck and chest (20), II–III	4 passes on high setting with 1440 nm and 4 passes on high setting with 1927 nm; 4 sessions at 4‐ to 6‐week intervals; 10% coverage	No AEs
Polder, 2024 [16]	Prospective, single‐center	Adults with mild‐to‐moderate photoaged skin^b^ (28; 89.3% female), I–V	4 passes on high setting with 1440 nm and 4 passes on high setting with 1927 nm (8 total passes per zone); 4 monthly sessions	Pain level range of 4.39–4.89^c^ within 60 min of treatment No serious AEs
Wang, 2024 [14]	Prospective	Adults seeking general facial resurfacing (14; 92.9% women), I–IV	4 passes on high setting with 1440 nm (energy 9 mJ; density 40 MTZ/cm^2^) and 4 passes on high setting with 1927 nm (energy 5 mJ; density 34 MTZ/cm^2^); 4 sessions at 2‐ to 4‐week intervals	Mild and transient roughness and dryness No serious AEs
1440‐nm handpiece				
Saedi, 2013 [17]	Prospective, single‐arm, nonrandomized	Adults (20; 19 female), I–IV and VI	Varied settings; average setting across all sessions was high (9 mJ) 8 passes used for all facial regions 6 sessions at 2‐week intervals	Mean pain sensation of 4.6 ± 0.1^c^ during treatments Mean heat sensation of 1.80^d^ immediately after treatment Flaking and dryness in 1 (5%) and 2 (10%) participants, respectively, at 2‐week follow‐up No serious AEs
1927‐nm handpiece				
Brauer, 2015 [18]	Prospective, single‐center, nonrandomized	Female adults with facial photodamage (23), I–IV and VI	≤ 8 passes at a fixed energy level of 5 mJ; 4–6 sessions at 14 ± 3–day intervals Coverage (5%, 7.5%, or 10%) and number of passes dependent on skin type, indication, and treatment response	Mean pain score of 3.4 ± 2.0^c^ across 6 sessions Mild‐to‐moderate average heat sensation after treatment Flaking in 1 and 2 participants at 1‐ and 3‐month follow‐ups, respectively Transient perioral PIH in 1 participant with FST IV Allergic reaction to topical anesthetic and mild acneiform eruption that spontaneously resolved No serious AEs
Elford, 2012 [19]	Prospective, single‐site, randomized	Participants with photoaged facial skin (40), NR	5‐mJ pulse energy and 5%–10% coverage; 6 sessions Participants were randomly assigned to laser alone or laser plus topical aqueous antioxidant serum (containing vitamin C, vitamin E, and ferulic acid)	Heat sensation in 60% of participants at day 1 Erythema, edema, and heat sensation average duration shorter in the laser plus topical antioxidant group (5 days) versus laser alone group (7 days) No serious AEs
Polder, 2013 [20]	Postmarket surveillance evaluation, multicenter	Adults with mild‐to‐moderate photodamage and/or dyspigmentation (78), I–V	4 passes over each anatomical unit of the face (left cheek, forehead, right cheek, chin; treatments were performed using low, medium, or high settings [2.5%, 3.75%, or 5% coverage, respectively], depending on participant tolerance, at a fixed energy level of 5 mJ) 81% of participants were treated at a high setting 6 sessions at 2‐week intervals	1 participant removed from study due to hyperpigmentation after third session Mean pain score of 3.6 ± 1.7^c^ immediately after treatment (across 6 sessions) No serious AEs

Abbreviations: AE, adverse event; FST, Fitzpatrick skin type; MTZ, microscopic treatment zone; NFDL, non‐ablative fractional diode laser; NR, not reported; PIH, postinflammatory hyperpigmentation.

^a^ Transient and mild‐to‐moderate erythema, edema, and crusting are expected skin reactions to laser treatment and were excluded from the table.

^b^ Photoaging classification according to the Glogau Photodamage Scale.

^c^ Measured using a visual analog scale (0 = no pain, 10 = unbearable or worst pain).

^d^ Measured using a 0–3 scale (3 = severe).

Three studies recently reported safety and tolerability outcomes for concurrent use of both the 1440‐ and 1927‐nm handpieces during treatment sessions (Table 2) [14, 15, 16, 17, 18, 19, 20]. The studies had similar protocols, with four passes each of the 1440‐ and 1927‐nm handpieces per session, and four sessions at 2‐ to 6‐week intervals. Handpiece order was only specified for one of the studies, wherein participants were treated with the 1440‐nm handpiece first, followed by the 1927‐nm handpiece [16]. Studies covered various treatment areas (i.e., neck, chest, and face) and FSTs ranging from I to V, although across studies, 79% of participants had FSTs II and III [14, 15, 16]. Expected skin reactions, such as erythema, edema, roughness, and dryness, were generally transient and mild to moderate in severity (on a 0–3 or 0–4 severity score, when measured) [14, 15, 16]. There were no serious AEs. A topical anesthetic of 7% lidocaine/7% tetracaine was applied 30 min before treatment, and pain levels were moderate and remained consistent throughout the treatment session [16]. Consistent tolerability and safety outcomes across studies suggest that concurrent use of the 1440‐ and 1927‐nm handpieces of the NFDL system is safe and tolerable with substantial reduction in posttreatment downtime compared to those for ablative fractional lasers [2, 15]. While these studies support a favorable safety profile, additional studies are warranted to include a larger number of participants and an even distribution of skin types.

A single study evaluated safety outcomes of the 1440‐nm handpiece alone (Table 2) [17]. The average treatment setting was high (9 mJ). The range of FSTs included I to IV and VI, with 80% of participants having FSTs II and III. Expected skin reactions were generally mild to moderate (on a 0–3 severity score) and transient. No serious AEs were reported. Similar to pain sensation with the dual 1440/1927‐nm NFDL treatment, pain sensation during treatment with the 1440‐nm handpiece alone was 4.6 ± 0.1 (mean ± standard deviation; moderate pain) on a 1 to 10 scale (10 = worst pain). Anesthetic ointment containing 30% lidocaine was applied 30 min before treatment. The mean score for heat sensation was also moderate (1.8 on a 0–3 scale; 3 = severe). Posttreatment side effects included transient dryness and flaking.

Three studies evaluated the safety of the 1927‐nm handpiece alone (Table 2) [18, 19, 20]. Treatment was administered at a fixed energy level (5 mJ), with coverage dependent on participant response. Of 23 participants in one study, 22 (96%) had FSTs ranging from I to IV and 1 (4%) had FST VI [18]; in another study, FSTs ranged from I to V, with 76% of participants having FSTs II and III [20]. Expected skin reactions, including erythema and edema, were mild to moderate (on a 0–3 severity scale, when measured) and self‐limited [18, 19, 20]. No serious AEs were reported. Mean pain sensation across sessions ranged from 3.4 ± 2.0 (with topical lidocaine/prilocaine, benzocaine/lidocaine/tetracaine, or lidocaine/tetracaine before treatment) to 3.6 ± 1.7 (without topical anesthetic) on a 1 to 10 scale [18, 20], and heat sensation was also mild to moderate [18]. One Asian participant with FST IV experienced transient perioral PIH, which resolved 2 months after study completion without medical intervention [18], and one participant (FST not reported) was removed from a study after three sessions owing to hyperpigmentation [20]. However, relatively high total treatment densities were used [20]. Similar to the results of treatment with the 1440‐nm handpiece, these studies demonstrate the overall safety and tolerability of the 1927‐nm handpiece.

### 3.2 Safety in Patients With Skin of Color

Patients with skin of color are more susceptible to certain AEs associated with laser resurfacing because of direct and indirect effects of treatment (e.g., melanosome disruption and postinflammatory effects, respectively) [4]. Risk of PIH is higher in patients with FSTs IV to VI compared to those with lighter skin types, and it can have a negative effect on psychological well‐being and quality of life [21]. In addition, laser treatments involving dermal injury pose a higher risk of keloid or hypertrophic scarring in patients of African or Asian ancestry compared to those with lighter skin [4]. Because melanin exhibits a light absorption spectrum from 250 to 1200 nm (visible and near‐infrared), non‐ablative fractional lasers in the mid‐infrared range (e.g., 1440 and 1927 nm) that target water rather than melanin are a safer option for the treatment of individuals with darker skin types [4, 22].

Four studies were identified that specifically investigated the safety and tolerability of the 1440‐ or 1927‐nm handpieces for skin resurfacing in participants with darker skin types (defined herein as FSTs III–VI; Table 3) [23, 24, 25, 26]. In studies using the 1440‐nm handpiece, settings were primarily high [24, 26] but were adjusted to medium or low in one study based on participant tolerance [24]. Studies involving the 1927‐nm handpiece used a fixed energy level (5 mJ) and 5% coverage in most cases [23, 25]. Expected skin reactions, including posttreatment erythema and edema, were mostly mild to moderate (on a 0–3 severity scale, when measured) and transient [24, 25, 26]. In one study, mild‐to‐moderate posttreatment erythema resolved within 12 h and was followed by mild peeling for 1 week or less [25]. Another study reported severe erythema once immediately after each session [24]. Treatment‐associated pain was variable, although the severity diminished over time, and most participants experienced mild‐to‐moderate heat sensation immediately after each session. Topical 5% lidocaine was applied 30 min before treatment. A third study reported a mean pain score of 3.6 ± 1.3 (mild to moderate on a 1–10 scale; 10 = worst pain; topical anesthesia protocol not described) and a mean heat sensation score of 1.1 ± 0.4 (mild on a 0–3 scale; 3 = severe) during and after treatment, respectively [26]. Notably, among the studies, only one transient case of isolated, localized hyperpigmentation was reported 2 weeks after the third session, which fully resolved 1 month after the final session [24]. No serious AEs and no worsening of preexisting conditions such as PIH or melasma were reported [23, 24, 25, 26]. These data suggest that treatments with the 1440‐ and 1927‐nm handpieces are generally well tolerated across diverse skin types even when used on high settings.

**TABLE 3:** Studies investigating the safety and tolerability of the dual 1440/1927‐nm NFDL for skin resurfacing in participants with skin of color (FSTs III–VI).

Author, year	Study design	Population (*N*), FST	Study procedures	Safety and tolerability outcomes^a^
Bae, 2020 [23]	Retrospective, single‐center	Adults with PIH (61; 56 women and 5 men), IV–VI	1927‐nm treatment with 5‐mJ fixed energy, 140‐μm spot size, 170‐μm depth, and 5% coverage 2 to > 5 sessions at monthly intervals Treatment areas included the face/cheeks (*n* = 42), legs (*n* = 8), or other (*n* = 11)	No AEs
Marmon, 2014 [24]	Prospective, single‐arm	Adults of Asian ancestry with facial photodamage (10), III–V	8 passes with 1440 nm High setting (9‐mJ energy; 390‐μm depth; density 50 MTZ/cm^2^) for most sessions; medium setting (7‐mJ energy; 340‐μm depth; density 40 MTZ/cm^2^); or low setting (4‐mJ energy; 280‐μm depth; density 40 MTZ/cm^2^) based on participant tolerance 4 sessions at 2‐week intervals	Severe erythema in 10% of participants immediately posttreatment for each session 1 case of isolated, localized hyperpigmentation 2 weeks after the third session that fully resolved 1 month after final session Severe and moderate treatment‐associated pain in 10% and 70% of participants after the final session, respectively; severity diminished as study progressed Some heat sensation during the procedure in most participants
Vanaman Wilson, 2018 [25]	Investigator‐blinded, single‐site, randomized	Adults with moderate‐to‐severe facial hyperpigmentation due to photodamage or melasma (40), III–V	8 passes with 1927 nm on high setting (140‐μm spot size, 170‐μm depth, 5‐mJ energy, 5% coverage), followed by randomized topical application (HQ 2% cream or bland moisturizer) 4 sessions at 2‐week intervals	Mild peeling for 1 week posttreatment No serious AEs
Wang, 2021 [26]	Prospective, interventional	Adults with rosacea and mild‐to‐moderate atrophic facial acne scars (30; 6 male and 24 female), III–IV	8 passes with 1440 nm on high setting (390‐μm depth, 4.5% coverage, 9‐mJ energy); 3 sessions at 4‐week intervals	Mean pain sensation of 3.6 ± 1.3^b^ during treatment Mean heat sensation of 1.1 ± 0.4^c^ after treatment Slight tightness and dryness in 3 and 1 participants, respectively, relieved with moisturizer No serious AEs

Abbreviations: AE, adverse event; FST, Fitzpatrick skin type; HQ, hydroquinone; MTZ, microscopic treatment zone; PIH, postinflammatory hyperpigmentation.

^a^ Transient and mild‐to‐moderate erythema, edema, and crusting are expected skin reactions to laser treatment and were excluded from the table.

^b^ Measured using a visual analog scale (0 = no pain, 10 = unbearable or worst pain).

^c^ Measured using a 0 to 3 scale (3 = severe).

## 4 Discussion

In this review, multiple studies demonstrate a favorable safety and tolerability profile for the dual 1440/1927‐nm NFDL system with concurrent use of both handpieces [14, 15, 16] or individual handpieces [17, 18, 19, 20, 26]. These studies report no serious AEs and mostly mild‐to‐moderate, expected skin reactions that typically resolved within a week without complications, including in participants with skin of color [14, 15, 16, 17, 18, 19, 20, 26]. Treatment was also well tolerated, with mild‐to‐moderate pain and heat sensations [16, 17, 18, 19, 20, 26].

These findings support the well‐established safety profile of NFDLs compared with ablative fractional lasers. Unlike ablative modalities, NFDLs create microscopic zones of dermal injury that preserve the epidermis and spare surrounding tissue, thereby minimizing postprocedural downtime [5, 6, 7]. As such, treatment with NFDL reduces wound‐related complications while achieving gentle therapeutic efficacy through controlled thermal injury, unlike ablative modalities that produce more dramatic results but require extended healing periods and greater risks of scarring, discoloration, and skin infections [3].

**FIGURE 3:**
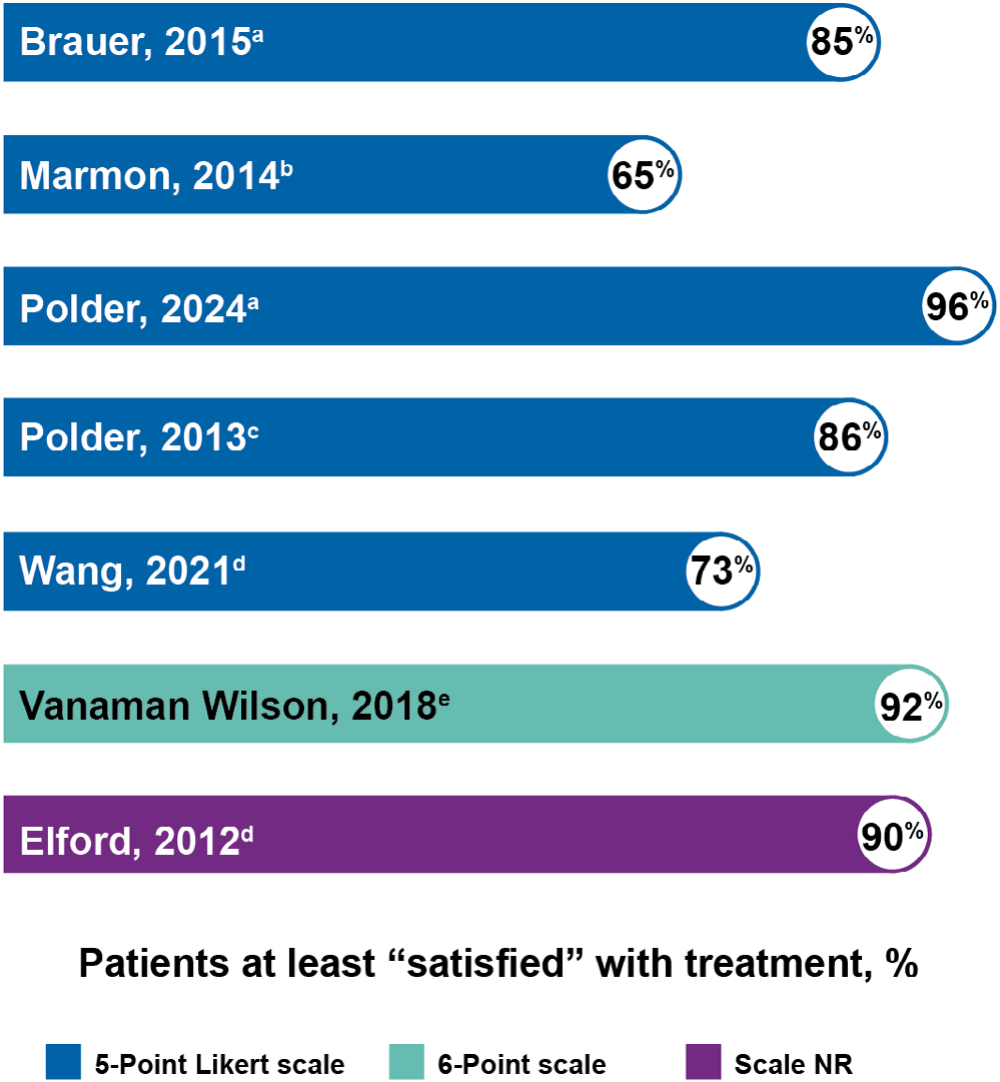
Studies that reported the percentage of participants who were at least “satisfied” with treatment with the dual 1440/1927‐nm NFDL or the individual handpieces [16, 17, 18, 19, 20, 24, 25, 26]. Most studies evaluated participant satisfaction with a 5‐point Likert scale (1 = very dissatisfied; 5 = very satisfied) or 6‐point Likert scale (1 = extremely satisfied, 6 = extremely dissatisfied) [16, 17, 18, 20, 24, 25, 26]. Saedi et al. 2013 reported an average satisfaction score on a 5‐point Likert scale at the last study visit (4.3 ± 0.7; *N* = 20) and was excluded from the graph [17]. ^a^At 3‐month follow‐up [16, 18]. ^b^Immediately after treatment [24]. Percentage is shown as an average calculated across 4 sessions. ^c^At 2‐week follow‐up [20]. ^d^Time point was not reported [19, 26]. ^e^Percentage is shown as an average of posttreatment weeks 4 and 12 [25]. NFDL, non‐ablative fractional diode laser; NR, not reported.

The favorable safety profile of the NFDL system is supported by high levels of participant satisfaction observed across multiple studies (Figure 3) [16, 17, 18, 19, 20, 24, 25, 26], with 65% to 96% of participants who were at least “satisfied,” [16, 18, 19, 20, 24, 25, 26] which significantly correlated with improvements in overall appearance (*p* ≤ 0.001) [17, 26]. The consistent achievement of high satisfaction rates across multiple studies underscores the system's ability to meet participant expectations while maintaining comfort and minimal side effects.

Because infrequent events of hyperpigmentation were reported in patients with FSTs III to V [18, 24], special considerations may be needed for the treatment of individuals with skin of color. Practitioners should carefully evaluate patient risk for PIH, including their history of PIH from previous procedures or treatments. Patients' skin can often be pretreated with topical lighteners (e.g., hydroquinone), treated concurrently or posttreatment with topicals (e.g., corticosteroids, hydroquinone), and/or treated immediately posttreatment with tranexamic acid topically and/or orally [21, 27, 28]. Patients with darker FSTs should be educated regarding photoprotection pretreatment and posttreatment, as well as sun avoidance in the immediate healing period [21]. Overall, in the clinical experience of the authors, the risk of PIH is low with these treatments when performed in appropriate patients and using suitable treatment settings.

This review has several limitations. The included studies exhibited considerable heterogeneity in terms of treatment parameters, participant populations, and outcome measures, making direct comparisons challenging. Many studies had relatively small sample sizes and lacked control groups, and the majority of articles lacked formalized statistical testing of safety outcomes. The assessment of participant satisfaction utilized different scales and methodologies, potentially affecting the comparability of reported satisfaction rates. Furthermore, limited data were available for certain skin types, particularly FSTs V to VI, which impacts the generalizability of findings across all skin phenotypes. Lastly, the majority of included studies (7/11) were industry‐sponsored by the device manufacturer, which may introduce bias in study design, outcome reporting, and interpretation of results, but also reflects the practical reality that most clinical research on proprietary devices is funded by the manufacturer.

In conclusion, this review demonstrates that the dual 1440/1927‐nm NFDL offers a tolerable treatment option for multiple esthetic concerns. The system's favorable safety profile, characterized by minimal postprocedural downtime and predictable posttreatment reactions, is reflected in consistently high participant satisfaction rates across studies. Treatment versatility is enhanced by the ability to use wavelengths individually or concurrently, allowing for customized treatment approaches. While further research with larger cohorts and standardized outcome measures would strengthen the evidence base, current data support the role of this NFDL system as a valuable tool in the esthetic practitioner's armamentarium for safe and effective skin resurfacing.

## Author Contributions

All authors meet the International Committee of Medical Journal Editors (ICMJE) criteria for authorship and have approved the final manuscript.

## Ethics Statement

The authors confirm that the ethical policies of the journal, as noted on the journal's author guidelines page, have been adhered to. No ethical approval was required as this is a review article with no original research data.

## Conflicts of Interest

R.G.G. and J.V.W. are investigators and consultants for Solta Medical. A.A.J. is an employee of Bausch Health Companies Inc. E.S.M. has no conflicts of interest to declare. K.D.P. is an investigator for Allergan, Bausch Health Companies Inc, and Galderma and has received honoraria and/or consultant fees from Allergan, Bausch Health Companies Inc, Galderma, and L'Oreal USA.

## Data Availability

Data sharing not applicable to this article as no datasets were generated or analyzed during the current study.
